# Hardware Implementation of a Fixed-Point Decoder for Low-Density Lattice Codes

**DOI:** 10.1007/s11265-021-01735-2

**Published:** 2022-01-31

**Authors:** Rachna Srivastava, Vincent C. Gaudet, Patrick Mitran

**Affiliations:** Department of Electrical and Computer Engineering, University of Waterloo, 200 University Ave. W., Waterloo, ON N2L 3G1 Canada

**Keywords:** Low-density lattice codes, Gaussian mixture, Fixed-point arithmetic, Serial and parallel FPGA architecture, Hardware architecture, Pipelining

## Abstract

This paper describes a field-programmable gate array (FPGA) implementation of a fixed-point low-density lattice code (LDLC) decoder where the Gaussian mixture messages that are exchanged during the iterative decoding process are approximated to a single Gaussian. A detailed quantization study is first performed to find the minimum number of bits required for the fixed-point decoder to attain a frame error rate (FER) performance similar to floating-point. Then efficient numerical methods are devised to approximate the required non-linear functions. Finally, the paper presents a comparison of the performance of the different decoder architectures as well as a detailed analysis of the resource requirements and throughput trade-offs of the primary design blocks for the different architectures. A novel pipelined LDLC decoder architecture is proposed where resource re-utilization along with pipelining allows for a parallelism equivalent to 50 variable nodes on the target FPGA device. The pipelined architecture attains a throughput of 10.5 Msymbols/sec at a distance of 5 dB from capacity which is a 1.8$\times$ improvement in throughput compared to an implementation with 20 parallel variable nodes without pipelining. This implementation also achieves 24$\times$ improvement in throughput over a baseline serial decoder.

## Introduction

Low-density lattice codes (LDLCs) are a special class of lattice codes proposed by Sommer et al. [1], whose construction and intended applications are substantially different from that of more familiar error-correcting codes such as low-density parity check (LDPC) codes, Polar, and Turbo codes. Lattice codes in general have shown great theoretical promise to exploit interference, possibly leading to significantly higher rates between users in multi-user networks. Research on LDLCs has concentrated on demonstrating the theoretically achievable performance limits of LDLCs, and until now there has been no reported hardware implementation, mainly due to the complexity of message-passing for LDLC decoding.

In this paper we investigate a field-programmable gate array implementation (FPGA) of a fixed-point decoder for low-density lattice codes. LDLCs are lattice codes whose construction was shown to allow for iterative decoding via message passing.

While linear error-correcting codes, e.g., LDPC and Polar codes, are based on finite fields, lattice codes are their Euclidean-space analog. In binary linear error-correcting codes, bit sequences are encoded into binary codewords before modulation, and the modulo-2 sum of any two binary codewords is again a binary codeword. In contrast, a lattice code directly outputs a point (i.e., lattice point) in Euclidean space, and the real-vector sum of two lattice points is again a lattice point, i.e., lattice codes have algebraic structure. Lattice codes have been shown to be effective for applications such as mitigating multi-user channel interference using the compute and- forward framework, and dirty-paper coding, by exploiting a code structure that is not present in error correcting codes [2–8].

LDLCs have a sparse (low-density) *H* matrix (inverse generator matrix) that renders iterative decoding (also called message-passing) an efficient decoding method for LDLCs [1, 9]. Despite this, implementation of the algorithm presented in [1] is not practical, either in software or hardware. This is primarily due to the fact that when the channel is additive-white-Gaussian-noise (AWGN), the messages exchanged between check and variable nodes in the iterative decoding algorithm are continuous functions, i.e., Gaussian mixtures. This is in contrast to many decoding algorithms for error-control codes, e.g., LDPC decoders, where messages can readily be reduced to single numbers such as log-likelihood ratios.

In prior work on LDLCs [1, 10–13], the continuous functions are either sampled and quantized or represented as Gaussian mixture messages denoted by parametric lists. In [1], each message is quantized to 1024 samples, which provides good accuracy in decoding but results in large storage and computational load. In [10, 12, 13], the messages are represented by Gaussian parametric lists of means, variances and coefficients. Nevertheless, as the decoding iterations progress, the number of components in the Gaussian mixtures grows exponentially and the implementation eventually has extremely large storage requirements and computational cost. To limit the number of components in the mixtures, Gaussian reduction algorithms are used to reduce the size of the messages after each decoding iteration. These methods reduce the message size significantly; however, even with all these reduction techniques, LDLC decoding is costly.

The emphasis of the literature to date in [1, 9–17] is on demonstrating theoretically achievable performance limits of LDLCs; not much work has been done towards a hardware implementation of LDLC decoding. Our work contributes in this direction with the aim to achieve a hardware implementation of a decoder for LDLCs. Several approximations are required to make this decoder feasible in hardware. However, these could result in a loss of performance.

In this work, the messages exchanged in iterative decoding are reduced to a single Gaussian using a moment-matching method in each decoding iteration [14]. Thus, only the mean and variance of a single Gaussian is exchanged between a check node and a variable node at each iteration. Since integer computations are inherently simpler than floating-point operations, a fixed-point arithmetic implementation is preferred. An important aspect of a fixed-point implementation is to determine the minimum number of bits for the required range and precision. A detailed quantization study is presented to find this minimum word length for fixed-point arithmetic. Efficient numerical techniques are then applied to approximate the required non-linear functions (division and exponentiation).

Previously we reported a serial LDLC decoder in FPGA, and in order to exploit the parallelism of iterative decoding, several parallel message computation blocks were included in the decoder [18]. Here we present a novel pipelined approach to implement the single-Gaussian LDLC decoder. With this design we achieve more than $\sim$ 24$\times$ improvement in throughput over the two-node serial implementation.

The outline of this paper is as follows. Section 2 defines lattice codes and LDLCs, and describes the properties and constraints for the LDLC *H* matrix. In Section 3, the iterative decoding algorithm is presented for a single-Gaussian decoder. Section 4 presents the implementation details of the single-Gaussian LDLC decoder in fixed-point arithmetic. Specifically 4.2 provides details of the quantization study and simulation results, followed by 4.3, which provides the key aspects of the decoder architecture, FPGA hardware implementation and results. Conclusions are provided in Section 5.

## Basic Definitions

Below, we provide a definition of LDLCs and some performance limits.

### Definition 1

An *n*-dimensional lattice, $\Lambda$ $\subset$ $\mathbb {R}^n$, is defined as all the integer linear combinations of *n* given linearly independent basis vectors, ${\underline{g}}_1,\ldots , {\underline{g}}_n \in \mathbb {R}^n$. Taking the basis vectors as the columns of the generator matrix *G*, (i.e. $G= ({\underline{g}}_1,\ldots , {\underline{g}}_n))$ the lattice $\Lambda$ is given by

1 $$\begin{aligned} \Lambda = \{ \underline{x} \in \mathbb {R}^n : \underline{x} = G\underline{b},\;\underline{b} \in \mathbb {Z}^n \}. \end{aligned}$$

### Definition 2

A low-density lattice code is an *n*-dimensional lattice code defined by a non-singular generator matrix that satisfies the condition that the constraint matrix, $H={G}^{-1}$, is sparse.

Following [1], the *H* matrix is chosen to be a regular Latin-square matrix, i.e., a matrix where every row and column has the same degree, *d*, of non-zero values ${\bar{h_1}}, {\bar{h_2}}, \ldots , {\bar{h_d}}$ except for possible sign flips and change of order. The sorted sequence of values ${\bar{h_1}} \ge {\bar{h_2}} \ge \ldots \ge {\bar{h_d}} > 0$ is termed as the generating sequence.

In [19], Poltyrev suggested a generic definition of capacity for lattice codes with no power restriction. According to this, capacity for lattice codes is defined as the maximal possible codeword density that can be recovered reliably at the receiver. This generalized capacity implied that there exists a lattice *G* of high enough dimension *n* that enables transmission with arbitrarily small error probability, if and only if the channel noise variance,

$$\begin{aligned} \sigma ^2 < {\root n \of {|\det (G) |^2}}/{2\pi e}, \end{aligned}$$

where $e = 2.71828...$ is Euler’s number (also known as the natural constant).

Thus the maximal performance limit for the lattice codes can be given by $\sigma ^2 = {\root n \of {|\det (G) |^2}}/{2\pi e}$.

Since for the designed LDLCs, $|\det (G) |= 1$, the theoretical noise (performance) limit is $\sigma ^2 = {1}/{2\pi e}$. For an AWGN channel without power restrictions, it is possible to quantify distance from capacity as the distance of the noise variance $\sigma ^2$ from ${1}/{2\pi e}$. To compute this, we first take the ratio of $\sigma ^2$ and ${1}/{2\pi e}$, i.e., $\sigma ^2/({1}/{2\pi e})$ and then convert the ratio to decibels (dB). All decoder frame error rate (FER) performance curves in this paper are thus measured with respect to the distance from capacity, $-10 \log _{10} (2\pi e \sigma ^2)$ (dB).

For this work, an *H* matrix of degree 3 is generated with the sequence $\{1, \frac{1}{\sqrt{3}}, \frac{1}{\sqrt{3}}\}$, and further normalized to obtain $\root n \of {|\det (H) |} =1$. The *H* matrix construction follows the algorithm described in [1].

## LDLC Iterative Decoding Algorithm

The sparse nature of the bipartite graph corresponding to the *H* matrix makes iterative message passing the preferred method for decoding LDLCs [1]. The AWGN channel model for LDLCs is given as

$$\begin{aligned} \underline{y} = \underline{x} + \underline{z}, \end{aligned}$$

where $\underline{x}$ is the transmitted lattice codeword (i.e., $\underline{x}=G\underline{b}$) , $\underline{y}$ is the received noisy message and $\underline{z}$ is a vector of independent and identically distributed (i.i.d.) Gaussian noise samples with common variance $\sigma ^2$.

In the parametric LDLC decoders, lists of means, variances and coefficients corresponding to the Gaussian mixture messages are exchanged between check nodes and variable nodes during the iterative decoding process [10, 12, 14]. For the single-Gaussian LDLC decoder implemented in this work the mixture messages are reduced to a single-Gaussian and only the mean and variance are exchanged.

A Gaussian mixture, *GM*(*t*), with *N* components is defined by

2 $$\begin{aligned} GM(t) = \sum _{k=1}^N \frac{c_k}{\sqrt{2\pi V_k}} e^{- \frac{(t-m_k)^2}{2V_k}}, \end{aligned}$$

where $$m_k$$, $$V_k$$ and $$c_k$$ are, respectively, the mean, the variance, and the mixing coefficient/weight of the $$k^{th}$$ component. A Gaussian mixture can then be efficiently represented by a set of triples $$\{(m_1$$,$$V_1,$$ $$c_1),$$ $$\ldots ,$$ $$(m_N,V_N,c_N)\}$$. If the coefficients sum to 1, i.e., $$\sum _{k=1}^N c_k = 1$$, then the Gaussian mixture is normalized. A single Gaussian is a special case of a Gaussian mixture when $$N=1$$, and can therefore be represented by the triple (*m*, *V*, *c*). If the single Gaussian is normalized, i.e., $$c=1$$, then this can be reduced to the mean-variance tuple (*m*, *V*).

Some intermediate steps in the single-Gaussian LDLC decoder generate Gaussian mixtures; however these mixtures are reduced to a single normalized Gaussian before message passing. Thus only mean-variance pairs are exchanged as messages between check nodes and variable nodes.

The basic steps of the iterative decoding algorithm for a single-Gaussian decoder are summarized below.

### Initialization

At the start of the decoding process, each variable node $x_k$ receives the single-Gaussian message from the AWGN channel given by $(y_k,\sigma ^2)$. Here $y_k$ is the mean and $\sigma ^2$ is the variance of the single Gaussian. This initial message is sent along all the edges connected to this variable node.

### Basic Iteration: Check Node Message

**Figure. 1:**
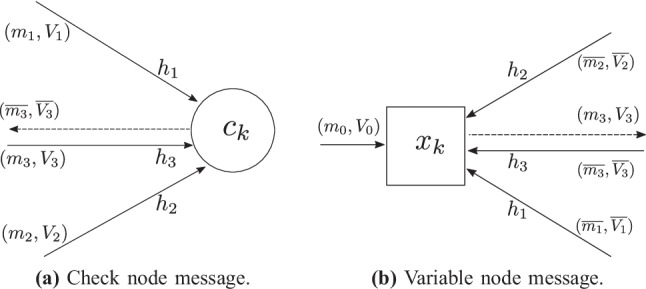
Illustration of all the incoming messages and the outgoing message along the edge with weight $h_3$ at (**a**) check node and (**b**) variable node.

Each check node has *d* input messages coming along the edges connected to it with weights $h_p$, $p = 1, \ldots , d$ as shown in Fig. 1a where $h_p$ is one of the ${\bar{h}}$’s with a possible sign flip.

The incoming messages are single Gaussians given by $(m_{\ell },V_{\ell })$, where $\ell = 1, 2 \ldots , d$. The mean of the outgoing check node message along the edge with weight $h_p$ is obtained by first multiplying for $\ell \ne p$, the mean of the $\ell ^{th}$ message with $\frac{h_\ell }{h_p}$, then summing the results over $\ell \ne p$ and a sign flip. The variance of the outgoing check node message along the edge with weight $h_p$ is obtained by first multiplying for $\ell \ne p$, the variance of the $\ell ^{th}$ message with $\frac{h_{\ell }^2}{h_p^2}$, then summing the results over $\ell \ne p$. The outgoing message is therefore the single-Gaussian $(\overline{m_p}, \overline{V_p})$ given by,

3 $$\begin{aligned} \overline{m_{p}}&= -\sum _{\ell \ne p} \frac{h_\ell m_{\ell }}{h_p}, \end{aligned}$$

4 $$\begin{aligned} \overline{V_{p}}&= {\sum _{\ell \ne p} \frac{h_\ell ^2V_{\ell }}{h_p^2}}. \end{aligned}$$

### Basic Iteration: Variable Node Message

As shown in Fig. 1b, each variable node receives *d* single-Gaussian messages along its edges denoted by $(\overline{m_{\ell }},\overline{V_{\ell }})$ for $\ell = 1, 2 \ldots , d$.

There are two primary steps performed at the variable nodes, a 1) periodic extension step and a 2) product step.

1. The periodic extension step generates periodic Gaussian mixtures from the incoming messages. In [1], this step is performed as a part of check node operations and the variable node receives the periodically extended Gaussian mixtures. However, in the single Gaussian decoder, the messages received from the check nodes are single-Gaussian messages and the periodic extension step occurs at the variable nodes [14]. This significantly reduces the storage requirements for the check node messages. In the periodic extension step, the mean of the incoming check node message along an edge with weight $h_l$ is first periodically extended as below,
  5 $$\begin{aligned} \overline{m_\ell }(i)&= \overline{m_\ell } + \frac{i}{h_\ell }, \end{aligned}$$
  where *i* denotes the $i^{th}$ extension. In principle, the variable *i* can take any integer value, but in practice the range is restricted so that the Gaussian components are near the channel message. This restriction is reasonable as the channel message is close to zero when evaluated far from its mean.
2. The outgoing variable node message along the edge with weight $h_p$ is computed by taking the product of the channel message, denoted by $(m_{0},V_{0})$, and all the Gaussian mixtures, except the mixture associated on that edge. This is then further reduced to a single-Gaussian using the second moment-matching-method [20].The product of two Gaussian mixtures is calculated by the pair-wise multiplication of each possible pair of components between the two mixtures. The product of two Gaussians is a scaled Gaussian. If two Gaussian components with triples $(\tilde{m}_1, \tilde{V}_1, \tilde{c}_1)$ and $(\tilde{m}_2,\tilde{V}_2,\tilde{c}_2)$ are multiplied, the resultant Gaussian is given by the triple $(m_F,V_F,c_F)$ calculated as,
  6 $$\begin{aligned} V_F&= \frac{\tilde{V_1} \tilde{V_2}}{\tilde{V}_1+ \tilde{V}_2}, \end{aligned}$$
  7 $$\begin{aligned} m_F&= V_F\Big (\frac{\tilde{m}_1}{\tilde{V}1} + \frac{\tilde{m}_2}{\tilde{V}_2}\Big ), \end{aligned}$$
  8 $$\begin{aligned} c_F&= \frac{\tilde{c}_1 \tilde{c}_2}{\sqrt{2\pi (\tilde{V}_1+\tilde{V}_2)}}e^{\frac{-(\tilde{m}_1 - \tilde{m}_2)^2}{2(\tilde{V}_1 + \tilde{V}_2)}} . \end{aligned}$$

### Final Decision

After every iteration, we estimate the decoded integer vector $\hat{\underline{b}}$. To get $\hat{\underline{b}}$, first an estimate $\hat{w}_k$ of the transmitted codeword element ${x_k}$ for $k = 1, 2 \ldots , n$ is computed. The variable, $\hat{w}_k$ is the mean of the single Gaussian obtained after the multiplication of the channel message and all the incoming check node messages (without omitting any) at each variable node (as described in Section 3.3) and the moment matching step [14].

Then $\hat{\underline{b}}$ is estimated as

9 $$\begin{aligned} \underline{\hat{b}} = \lfloor {H\cdot \underline{{\hat{w}}}}\rceil , \end{aligned}$$

where $\lfloor \rceil$ denotes coordinate-wise integer rounding [21].

The decoded integer vector, $\hat{\underline{b}}$, is computed after every decoding iteration and the iterative decoding process is terminated as soon as decoding is successful. Early stopping reduces the average number of iterations required for decoding and is commonly used in iterative decoding [17, 22–25].

## LDLC Decoder Implementation

The product-step at the variable node generates a Gaussian mixture that must be reduced to a single Gaussian before it can be sent along an outgoing edge of the node. The single Gaussian approximation for the Gaussian mixture is computed using the second-moment-matching method, now described below.

For a Gaussian mixture message denoted by triples of mean, variance and mixing coefficients, i.e., by $$\{(m_1$$,$$V_1,$$ $$c_1),$$ $$\ldots ,$$ $$(m_N,V_N,c_N)\}$$, the second-moment-matched single Gaussian, $$(m_\mathsf{MM},V_\mathsf{MM})$$ is obtained by first normalizing the mixture according to $$r_k = c_k/(\sum _{k=1}^{N}c_k)$$, and then parameters $$m_\mathsf{MM}$$ and $$V_\mathsf{MM}$$ are calculated as

10 $$\begin{aligned} \begin{aligned} m_\mathsf{MM}&= \sum _{k=1}^{N}r_km_k, \\ V_\mathsf{MM}&= \sum _{k=1}^{N}(r_kV_k + r_k(m_k - m_\mathsf{MM})^2). \end{aligned} \end{aligned}$$

For improved numerical stability, at the variable nodes, the smallest allowable variance is limited to a certain minimum value denoted $\mathsf {minvar}$. In the literature a $\mathsf {minvar}$ of $0.03\sigma ^2$ was adopted [12]; however, based on our simulations, $\mathsf {minvar}$ can be increased to $0.1\sigma ^2$ without any loss in decoder performance, where $\sigma$ is the standard deviation of the received Gaussian channel message. In this work, any variance less than $0.1\sigma ^2$ is increased back to $0.1\sigma ^2$.

Moreover, all variances in this implementation are measured relative to the channel noise variance, e.g., for $V=2$ in the implementation the actual variance is $2\sigma ^2$.

Simulation results presented for a single-Gaussian decoder are for random lattice codewords in the integer range $\underline{b} \in \mathcal{{L}}^n$, where $\mathcal {L}$ = {−2, −1, 0, 1, 2}.

### Number of Decoding Iterations

In [1, 11, 12, 14], the reported performance results for the LDLC decoder are for 200 decoding iterations. However, in order to obtain reasonable decoding latency as well as limit power consumption, fewer decoding iterations are preferred. Therefore finding a suitable number of decoding iterations is an important step towards a feasible hardware.

**Figure. 2:**
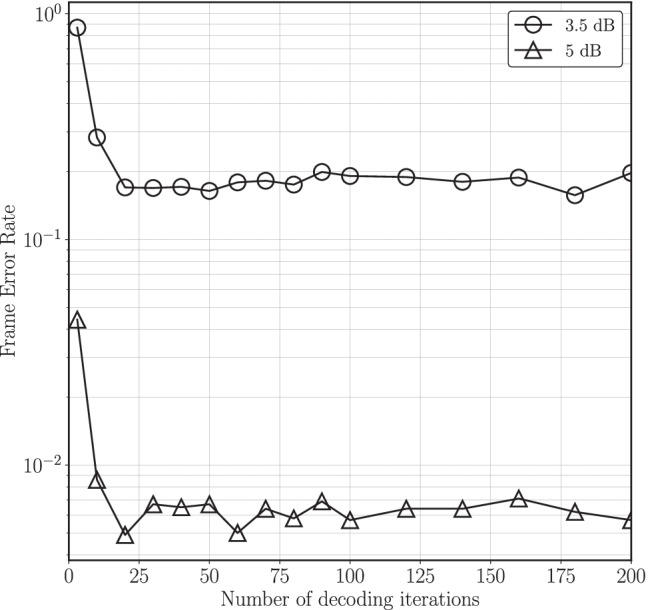
Frame error rate performance for different number of decoding iterations at distance from capacity of 3.5 dB and 5 dB.

Figure 2 shows the decoder performance versus number of decoding iterations at a distance from capacity of 3.5 dB as well as 5 dB. As the graph suggests, with 20 decoding iterations, the decoder can achieve comparable performance to 200 decoding iterations, but in significantly less run time.

### Fixed-Point Quantization Study

In the design space of this work, a fixed-point arithmetic is sufficient to implement the decoder in hardware (demonstrated further in the section). However, a key aspect of fixed-point arithmetic is to determine the range and precision requirements of the design.

In fixed-point representation, every number has a fixed word length of *W* bits that consists of $W_i$ integer bits, $W_f$ fractional bits and a sign bit. In this paper fixed-point representations are denoted by Q$W_i.W_f$, e.g., Q10.8 indicates 10 bits to represent the integer range and 8 bits for the fractional precision, and one sign bit (19 bits total).

**Figure. 3:**
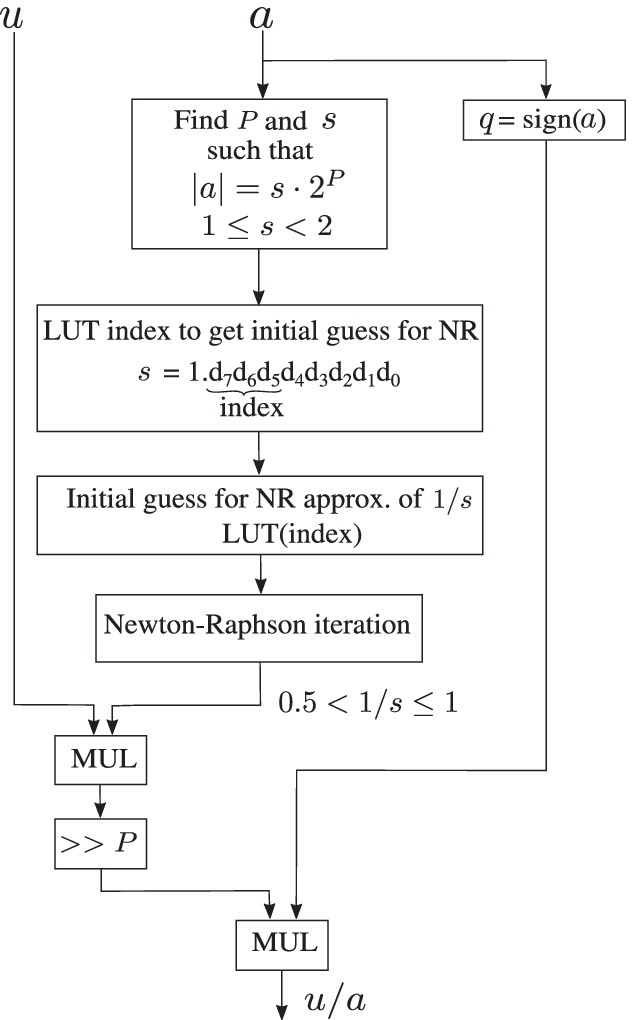
Flow-chart for the division function approximation in fixed-point arithmetic using Newton-Raphson (NR) method, used at the variable nodes.
    Simulations were performed to find an optimal LUT size to get a reasonable initial guess for the NR approximation and ensure high accuracy of the division result with a minimum number of iterations. Specifically the performance with LUT sizes of 4, 8 and 16 entries were computed by numerical simulation using the procedure described below. For LUT sizes of 8 and 16 entries, similar FER performance is obtained after two NR iterations while one NR iteration results in performance loss compared to 2 iterations. For a LUT size of 4 entries, FER performance is 0.2 dB worse than that of the 8 entry LUT even after 2 or more iterations. Based on these results, we create a LUT with only 8 numerical values.
    In order to obtain these 8 initial values, the range of *s*, i.e., 1 to 2, is divided into 8 equal sub-intervals: 1, ${1}\frac{1}{8}$, ${1}\frac{2}{8}$, ${1}\frac{3}{8}$, ${1}\frac{4}{8}$, ${1}\frac{5}{8}$, ${1}\frac{6}{8}$, ${1}\frac{7}{8}$, 2. Then, the mid-points of these sub-intervals are computed. As we want to calculate the reciprocal of *s*, the mid-points of these sub-intervals is obtained by their geometric means. For example, the geometric mean of ${1}\frac{1}{8}$ and ${1}\frac{2}{8}$ is $({1\frac{1}{8}\times 1\frac{2}{8}})^{1/2}$. Then we compute the reciprocal of this geometric mean, i.e., $({1\frac{1}{8}\times 1\frac{2}{8}})^{-1/2}$.
    In a similar fashion, the other entries of the look up table are computed, i.e., $({1\times 1\frac{1}{8}})^{-1/2},$ $({1\frac{1}{8}\times 1\frac{2}{8}})^{-1/2},\ldots , ({1\frac{7}{8}\times 2})^{-1/2}$, and converted to a fixed-point representation that is used for the rest of the decoder.
    For a fixed-point number, *s*, we use the 3 bits after the leading 1 (since $1 \le s < 2$) as the index for the LUT to obtain the initial guess. The complexity of this method is constant time, i.e., $\mathcal {O}(1)$ [26].
  - *Approximation of exponential function using LUTs:* A direct implementation of the exponential function in FPGA has large resource requirements and design complexity. However LUT-driven methods make an exponential implementation feasible in limited FPGA resources.
    In an LDLC decoder implementation, the exponent is always non-positive. Specifically, we approximate $\exp (-a/2)$ for $a \ge 0$, where the division by two accounts for the factor of $\frac{1}{2}$ in the exponent of (8).
    For ease of computation, the exponential function $\exp {(-a/2)}$ is written as the product of three easily computable terms.
    In particular, *a* is decomposed into 3 parts as
    13 $$\begin{aligned} {a}&= {I_2}2^{P_2} + {I_1}2^{P_1} + {I_0}2^{P_0}, \end{aligned}$$
    where $P_0<P_1<P_2$ are the positions of the least significant bit of each part and $I_0,I_1,I_2$ are integers that depend on *a* such that $0 \le I_0 < 2^{P_1-P_0}$, $0 \le I_1 < 2^{P_2-P_1}$ and $0 \le I_2 < 2^{W_i-P_2}$. Figure 4 illustrates the relationship between *a* and $I_0,I_1$ and $I_2$. Since $I_0$ is comprised of $P_1-P_0$ bits, its range is from 0 to $2^{(P_1-P_0)}-1$. Likewise $I_1$ is comprised of $P_2-P_1$ bits and its range is from 0 to $2^{(P_2-P_1)}-1$ and $I_2$ comprises of $W_i-P_2$ bits with its range from 0 to $2^{(W_i-P_2)}-1$.

1. *Approximation of non-linear functions:* The fixed-point implementation has two non-trivial non-linear functions: division and exponentiation.
  - *Approximation of division function using Newton-Raphson method:* A straightforward method to approximate division in fixed-point is integer long division. However, integer long division computation, i.e., $\mathtt {Qdiv}(u,a)~=~{(u \ll W_f)}/{a}$ can be expensive in terms of time and hardware. As an alternative, $\mathtt {Qdiv}$ can also be implemented as
    11 $$\begin{aligned} \mathtt {Qdiv}(u,a)&= \mathtt {Qmul}(u,\mathtt {NR\_{reciprocal}}(a)) , \end{aligned}$$
    where $\mathtt {NR\_{reciprocal}}(a)$ is the reciprocal of *a* calculated using the Newton-Raphson (NR) method, which is then multiplied with *u* using the fixed-point multiplication function, $\mathtt {Qmul}$.
    For the Newton-Raphson method, convergence to the correct solution depends critically on a reasonable initial guess. In a fixed-point decoder, this initial guess is obtained using a look-up table (LUT). To reduce the look-up table size and minimize approximation errors, we do not approximate the reciprocal of *a*, but instead, the fixed-point number *a* is written as $q \times (s\cdot 2^P)$ where *P* is an integer, *q* is $\pm 1$ and *s* is a non-negative fixed-point number with $1 \le s < 2$. The reciprocal of *s* is then calculated using $\mathtt {NR\_reciprocal}$. This reciprocal is multiplied with *u*, scaled back by $2^{-P}$ and further multiplied with *q* to get the value of *u*/*a*.
    In this method, the reciprocal of *s* is always in the range $0.5 < {1}/{s} \le 1$, which can be represented precisely enough with a small number of fractional bits.
    The division function is thus implemented in the fixed-point LDLC decoder as (See Fig. 3),
    12 $$\begin{aligned} \mathtt {Qdiv}(u,a)&= q \times (\mathtt {Qmul}(u, \mathtt {NR\_reciprocal}(s)) \gg P). \end{aligned}$$

**Figure. 4:**
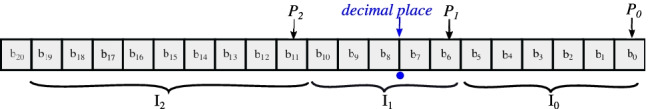
Diagram to show the relationship between *a* and $I_0$, $I_1$ and $I_2$ as used in the approximation of the exponential function in fixed-point arithmetic at the variable nodes (reproduced from [18]).
    Then the exponential is given as,
    14 $$\begin{aligned} \begin{aligned} \exp {(-a/2)}&= \exp {(-I_2 2^{P_2}/2)} \\&\quad \times \exp {(-I_1 2^{P_1}/2)}\exp {(-I_0 2^{P_0}/2)}. \end{aligned} \end{aligned}$$
    Decomposing *a* into three smaller parts thus allows for three smaller look-up tables instead of a single large lookup table to approximate the exponential.
    We choose $P_0$, $P_1$ and $P_2$ carefully, e.g., $P_0=-W_f$, $P_2$ is the smallest positive integer such that $\exp {(-2^{P_2}/2)}$ underflows the fixed-point representation of the LDLC decoder and $P_1 = \lfloor {(P_0+P_2)}/2\rfloor$. Due to the choice of $P_2$, if $I_2 > 0$ then $\exp {(-a/2)}$ is approximated as 0. Otherwise $I_2 = 0$ and thus $\exp {(-I_2 2^{P_2}/2)} = 1$ and only two small look-up tables are sufficient to compute $\exp {(-I_1 2^{P_1}/2)}$ and $\exp {(-I_0 2^{P_0}/2)}$.
    The first lookup table contains $2^{(P_2-P_1)}$ entries to approximate $\exp (-I_12^{P_1}/2)$ for possible $I_1$ values, i.e., 0 to $2^{(P_2-P_1)}-1$. Likewise, the second lookup table approximates $\exp (-I_02^{P_0}/2)$ for all possible $2^{(P_1-P_0)}$ values of $I_0$.
2. *Optimal word length and Newton-Raphson (NR) iterations for fixed-point decoder:* In order to find the optimal word length for the fixed-point representation, simulations are performed for different values of $W_i$ and $W_f$. Figure 5 compares the frame error rates for different values of $W_f$ while keeping $W_i$ large and varying the number of NR iterations for block length *n* = 1000. Figure 6 compares decoder performance for different values of $W_i$ while $W_f$ is fixed.

**Figure. 5:**
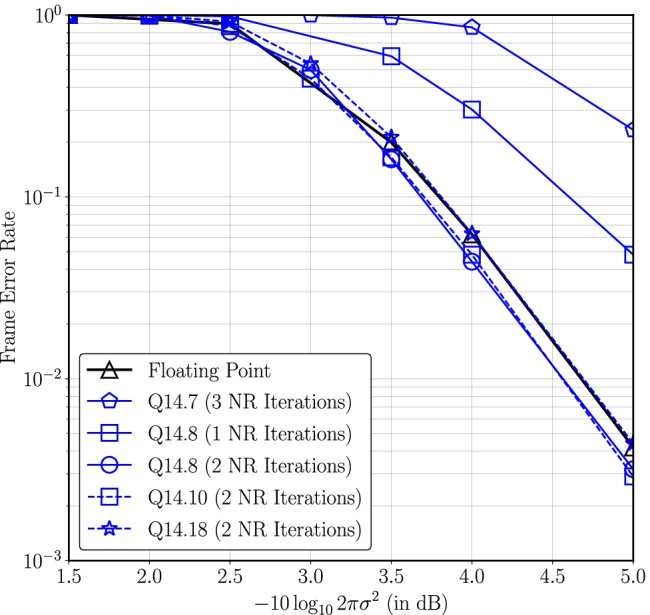
Frame error rate for different numbers of fractional bits and Newton-Raphson iterations for $n=1000$ and $W_i=14$ where $-10\log _{10}2\pi e\sigma ^2$ is distance from the theoretical noise limit [1] (reproduced from [18]).

**Figure. 6:**
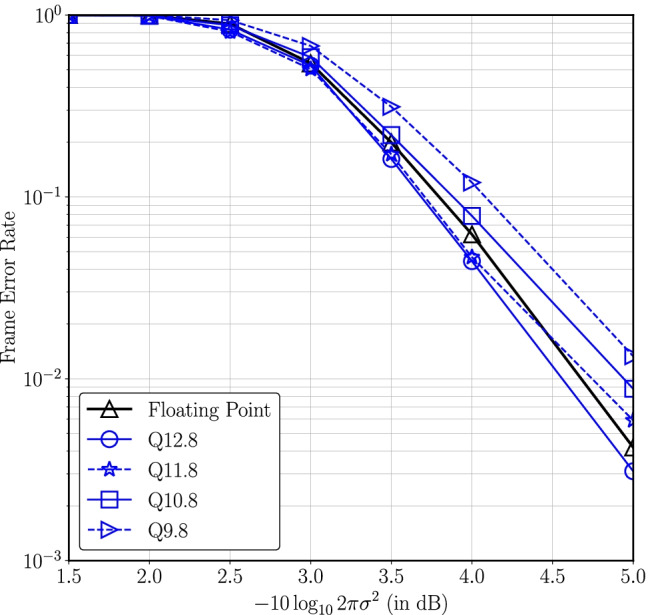
Frame error rate for different numbers of integer bits and two Newton-Raphson iterations with $n~=~1000$ and $W_f=8$ (reproduced from [18]).
  A key observation in Fig. 5 is that at 4.5 dB the FER for Q14.8 with 2 NR iterations is 0.13 dB better compared to Q14.18.
  The LDLC decoder is a sub-optimal decoder because it is both iterative and parametric in nature. Therefore, it is anticipated that some approximations could potentially improve the decoder performance.
  To understand this behaviour, simulations were performed with a floating-point decoder where the components of the Gaussian mixture message at the variable node that have coefficients less than a certain threshold, denoted $\mathsf {coeff^{th}}$, are removed from the Gaussian mixture. As illustrated in Fig. 7, the FER does not monotonically increase with $\mathsf {coeff^{th}}$, but instead achieves a minimum at approximately $\mathsf {coeff^{th}} \approx 0.03$. Based on these simulation results, an appropriate choice of $W_f$ helps the decoder by naturally underflowing the fixed-point representation of small coefficients. However if $W_f$ is further reduced, then performance deteriorates.

**Figure. 7:**
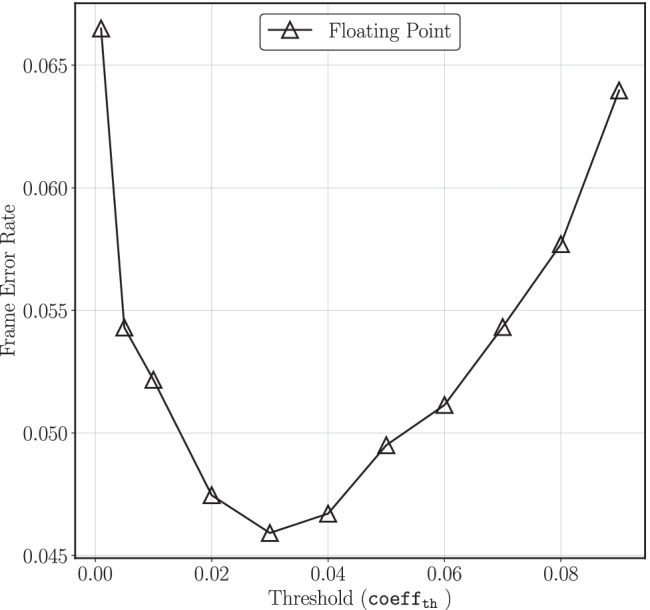
Effect of removing small coefficients from Gaussian mixture in floating point LDLC decoder at $-10\log _{10}2\pi e\sigma ^2$ = 4 dB, $n~=~1000$ (reproduced from [18]).
  A similar trend has previously been seen in published fixed-point Turbo decoders, where the quantization methodology leads to fixed-point implementations where the bit error rate (BER) can be slightly better than the BER of floating-point implementation [27].
  Figure 6 plots the effect of a different number of integer bits on the decoder performance. The results reported here demonstrate that the decoder performance degrades with smaller $W_i$ due to the computation errors that occur from the saturation in arithmetic operations, primarily multiplication.
  Based on the results in Figs. 5 and 6, a word length of 21 with 12 integer bits, 8 fractional bits and a sign bit is an appropriate choice for a fixed-point representation. As seen in Fig. 6 the single-Gaussian, Q12.8 (with 2 NR iterations) achieves an FER of $3\cdot 10^{-3}$ at a distance of 5 dB from capacity that is slightly better than the floating-point decoder.

### LDLC Decoder FPGA Implementation

We now present our FPGA implementation results including 3 architectures: A) an architecture with a single check node and a single variable node, B) an architecture where parallelism and hardware resources are exploited to implement 20 variable nodes and a single check node and C) an architecture with a single check node and with two-stage pipelining to achieve an effective parallelism equivalent to 50 variable nodes.

Architecture A) A single check node and a single variable node: A fully parallel LDLC decoder implementation is large and does not fit on the target reconfigurable device. However, there are possible approaches to build the complete decoder on a target FPGA device that can fit a few check and variable nodes.

**Figure. 8:**
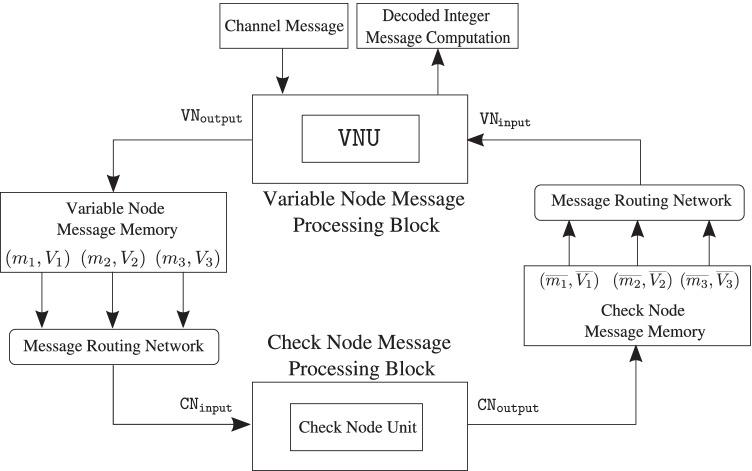
Block diagram of a two-node serial single-Gaussian LDLC decoder with one check node and one variable node (reproduced from [18]).

To better understand the issues involved in an LDLC decoder implementation and make key estimates, e.g., resource requirements and performance, as a baseline design Fig. 8 presents a serial architecture for the decoder. This implementation contains one check node and one variable node. The check node and variable node messages generated during decoding iterations are stored in two separate single-ported memory banks. Read-only-memories (ROMs) are used to store check node connections to variable nodes and vice-versa, according to the *H* matrix. The edge weights of the connections are stored in a separate ROM.

In order to compute the outgoing messages from a check node, $c_k$, the *message routing network* looks up the variable nodes connected to $c_k$ and the edge weights associated with these connections from the respective ROMs. Then, it fetches the corresponding means and variances from the *variable node message memory* and the *check node message processing block* computes the outgoing messages. The *variable node message processing block* receives the check node messages and computes the outgoing variable node messages in a similar fashion.

#### Check node message processing block

**Figure. 9:**
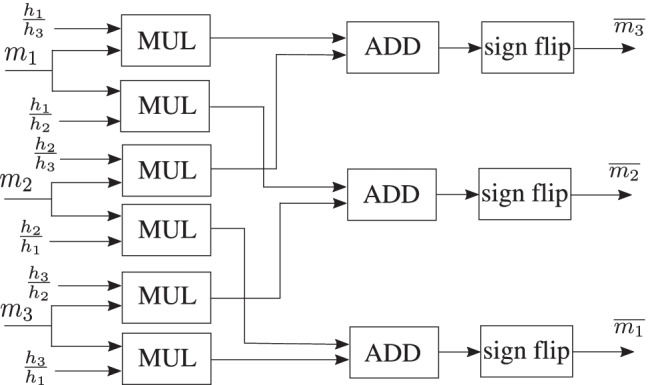
Block diagram for the mean computation of the outgoing messages at the check node. The mean is computed by first multiplying each incoming message with its respective edge weight (except the one on the outgoing edge), summing the results and further dividing the result of the summation by the outgoing edge weight along with a sign flip.

**Figure. 10:**
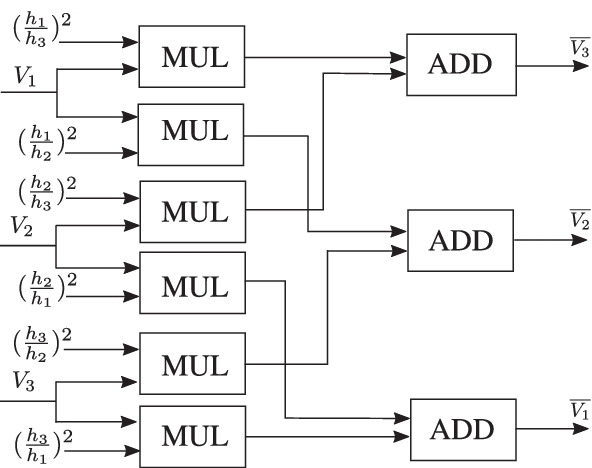
Block diagram for the variance computation of the outgoing check node messages.

**Figure. 11:**
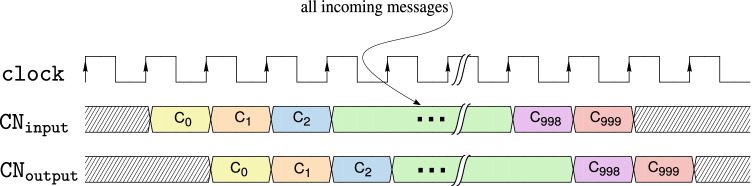
Timing diagram of the check node message processing block in architecture A , B and C.

The check node message processing block consists of check node unit that performs convolution of the incoming messages according to (3) and (4). Figures 9 and 10 show the mean and variance computations of the outgoing check node messages that can be implemented with only a few adaptive logic modules (ALMs), digital signal processing (DSP) blocks and registers. Figure 11 depicts the timing diagram for the check node message processing block in architectures A, B, and C.

#### Variable node message processing block

The variable node message processing block consists of a variable node unit, $\mathtt {VNU}$. As discussed in Section 3.3, at each variable node unit, $d-1$ periodically extended check node messages and the channel message are multiplied and the resulting product is reduced to a single-Gaussian using second moment-matching. To compute the variable node message efficiently, a forward-backward recursive algorithm is used [10].

The algorithm is initialized with the channel message. Let’s denote the periodically extended messages with ${\mathtt {MPeriodic_\ell }}$ where $\ell = 1, 2 \ldots , d$ and the Gaussian mixture reduction of Section 4 (including the normalization step) by $\mathtt {GMR}$.

The pseudo-code for this forward-backward recursion algorithm is given in Algorithm 1. Here “$\cdot$” denotes product of Gaussian mixtures as described in Section 3.3. 
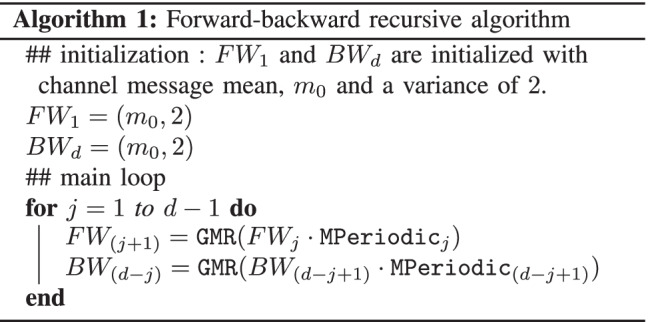


Once the forward-backward messages, $FW_\ell$ and $BW_\ell$ for $\ell = 1, 2 \ldots , d$ are computed, the outgoing variable node messages, i.e., $(m_\ell , V_\ell )$ for $\ell = 1, 2 \ldots , d$ are obtained as,

15 $$\begin{aligned} {(m_\ell , V_\ell )}&= FW_\ell \cdot BW_\ell . \end{aligned}$$

The estimate of the transmitted codeword, $\hat{w_k}$ is the mean of the computation, $\mathtt {GMR}(FW_2 \cdot BW_1)$.

**Figure. 12:**
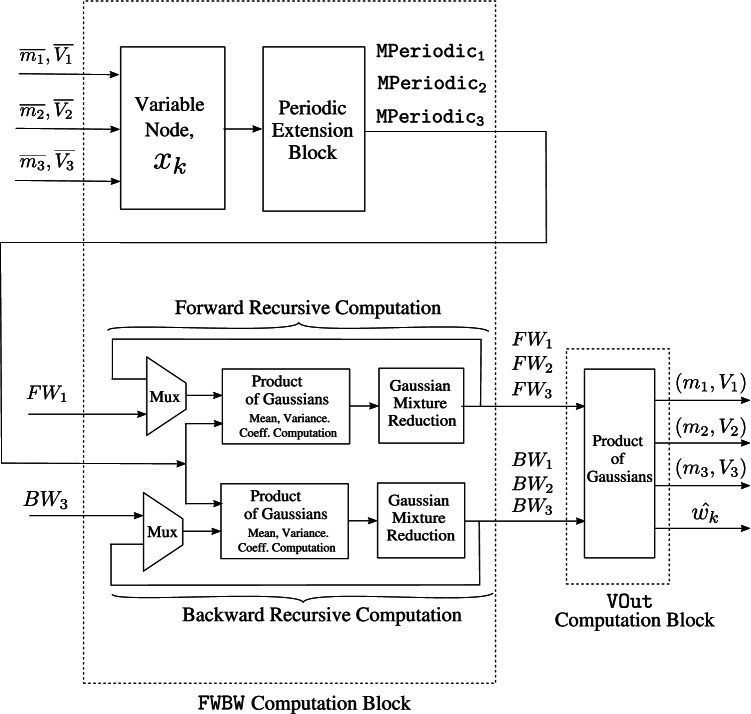
High-level architecture of a variable node unit ($\mathtt {VNU}$) for $d=3$. At a variable node, $x_k$, the incoming check node messages are periodically extended, $FW_\ell$ and $BW_\ell$ for $\ell = 1, 2 \ldots , d$ are computed in $\mathtt {FWBW}$ computation block and finally the outgoing variable node messages, $(m_\ell ,V_\ell )$ for $\ell = 1, 2 \ldots , d$ and estimate for transmitted codeword, $\hat{w_k}$; is obtained in the $\mathtt {VOut}$ computation block.

**Figure. 13:**
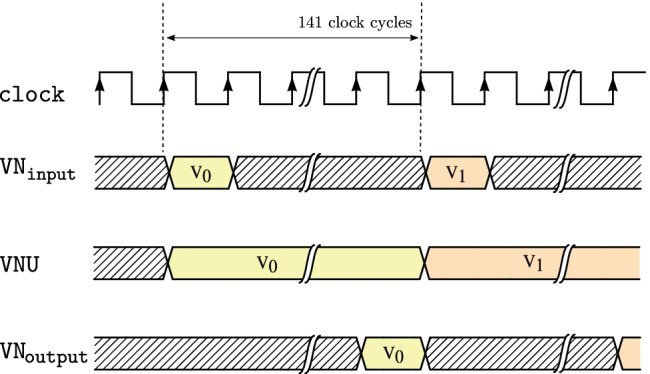
Timing diagram of the variable node message processing block in architecture A.

The top-level architecture of the variable node unit is presented in Fig. 12. The timing diagram for the variable node message processing block in architecture A is shown in Fig. 13.

The computation of $FW_j{\cdot }\mathtt {MPeriodic}_j$ in Algorithm 1 computes the product of a single Gaussian ($FW_j$) with a Gaussian mixture ($\mathtt {MPeriodic}_j$). The single Gaussian is normalized. Thus, it has a single component of weight ‘1’. The Gaussian mixture is obtained by periodically extending a normalized single Gaussian. Thus all the weights of the mixture are equal and are also ‘1’. In addition, all the variances of the mixture are equal to that of the single Gaussian that was periodically extended and hence, are all equal. Therefore, the term $\frac{\tilde{c}_1 \tilde{c}_2}{\sqrt{2\pi (\tilde{V}_1+\tilde{V}_2)}}$ in (8), which must be computed for each component in the product $FW_j{\cdot }\mathtt {MPeriodic}_j$, is the same.

Since the components in the product are explicitly normalized in the Gaussian mixture reduction step that follows the computation of the product, to reduce complexity, for the computation of the product $FW_j{\cdot }\mathtt {MPeriodic}_j$, the weights in (8) are instead replaced with

16 $$\begin{aligned} c_F&= e^{\frac{-(\tilde{m}_1 - \tilde{m}_2)^2}{2(\tilde{V}_1 + \tilde{V}_2)}} . \end{aligned}$$

Similarly, the weights in (8) are also replaced with (16) for the computation of the product $BW_{(d-j+1)} \cdot {\mathtt {MPeriodic}_{(d-j+1)}}$.

This serial implementation was designed as a proof-of-concept for LDLC decoding in hardware. However, more than one check node and/or variable node with design optimizations can provide considerable improvement in decoding speed.

Architecture B) A single check node and 20 variable nodes: The variable node unit described above requires 140 clock cycles for message computation while the check node takes a single cycle, and thus the variable node limits the throughput. Several parallel variable nodes can render variable-node message computation faster and boost decoder throughput significantly. To exploit the inherent parallelism of iterative decoding we implement 20 parallel variable nodes with the available resources on the target FPGA (of course, a larger FPGA could potentially fit even more variable nodes).

**Figure. 14:**
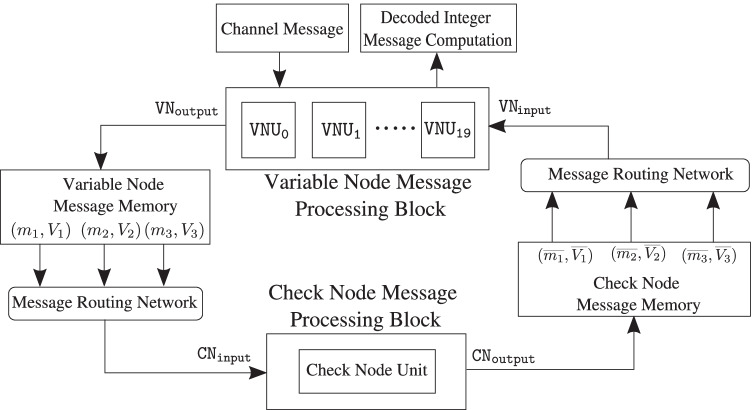
Top-level block diagram of the LDLC decoder with one check node and 20 parallel variable node units (reproduced from [18]).

**Figure. 15:**
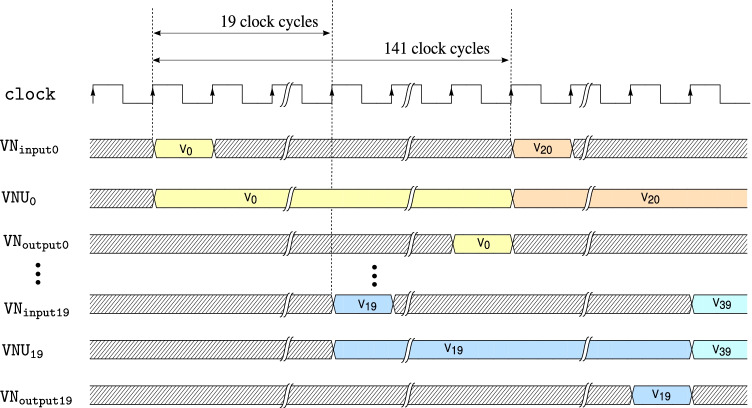
Timing diagram of the variable node message processing block in architecture B.

Figure 14 shows the decoder architecture where the check node message processing block has a single check node and the variable node message processing block contains 20 parallel variable node units denoted by $\mathtt {VNU_p}$, with inputs $\mathtt {VN_{input\{p\}}}$ and outputs, $\mathtt {VN_{output\{p\}}}$ for $p = 0, 1, 2 \ldots , 19$. Figure 15 shows the timing diagram for the *variable node message processing block* in architecture B. The *message routing network* fetches check node messages for one variable node every clock cycle and the incoming messages are driven to $\mathtt {VN_{input\{p\}}}$ for $p = 0, 1, 2 \ldots , 19$ in 20 clock cycles sequentially.

Architecture C) A single check node and with two-stage pipelining to achieve an effective parallelism equivalent to 50 variable nodes:

After additional data flow and design optimizations, in the variable node unit shown in Fig. 12, the $\mathtt {FWBW}$ computation block requires 109 clock cycles while the calculations in the $\mathtt {VOut}$ computation block take 10 clock cycles. This implies that one $\mathtt {VOut}$ computation block can be sufficient to process the output from 10 $\mathtt {FWBW}$ computation blocks (when pipelined), which could provide significant hardware savings.

For efficient variable node message computation, we implement a two-stage pipeline in the *variable node message processing block*. The first stage of the pipeline consists of 10 $\mathtt {FWBW}$ computation blocks that compute the $FW_\ell$ and $BW_\ell$ messages corresponding to 10 variable nodes, $x_k$ for $k = 0, 1, 2 \ldots , 9$, according to Algorithm 1. Further, the second stage block reads-in stage 1 output and computes outgoing variable node messages according to (15) corresponding to a variable node. The design components, primarily adders and multipliers, are reused in different clock cycles within the two pipeline stages. For convenience, this pipelined architecture is termed as $\mathtt {VNUCluster}$.

**Figure. 16:**
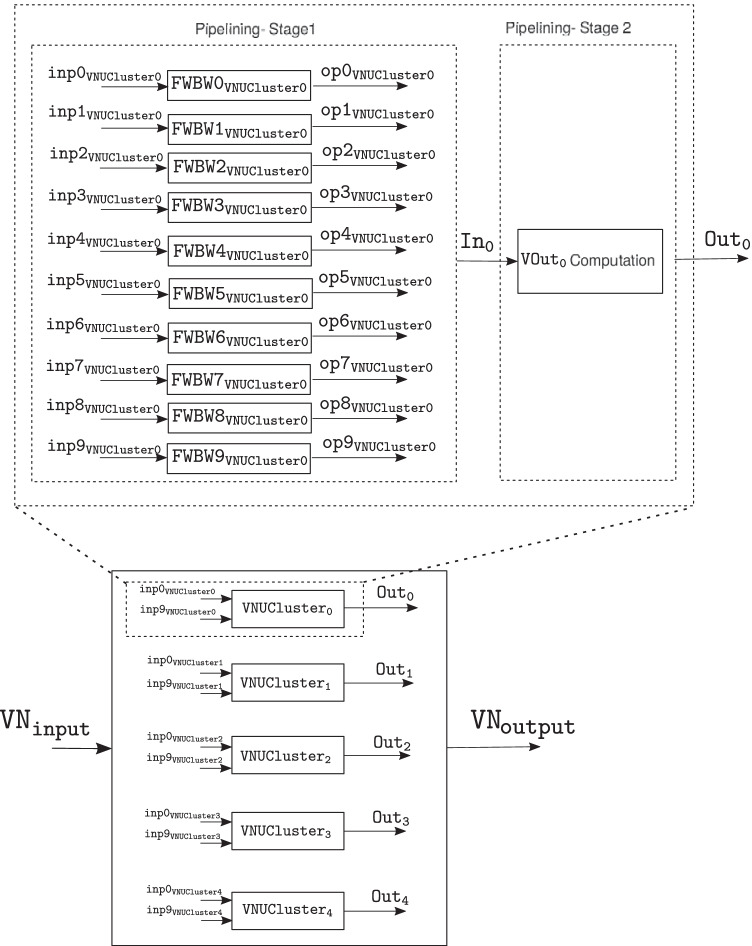
High-level diagram of the variable node message processing block used in architecture C, that consists of 5 $\mathtt {VNUCluster}$ blocks. The two stage pipelining used in $\mathtt {VNUCluster}$ blocks is shown specifically for $\mathtt {VNUCluster_0}$.

The resources on the target FPGA are sufficient to implement 5 parallel $\mathtt {VNUCluster}$ blocks ($\mathtt {VNUCluster_p}$ for $p = 0, 1, 2 \ldots , 4$), achieving a parallelism equivalent to 50 variable node units ($\mathtt {VNUs}$), thus rendering significantly reduced computation time for each variable node message generation overall. Figure 16 shows the top-level block diagram of the *variable node message processing block* used in architecture C that consists of 5 $\mathtt {VNUCluster}$ blocks.

The sub-blocks of the pipelining inside the $\mathtt {VNUCluster}$ blocks are shown specifically for $\mathtt {VNUCluster_0}$. Here, 10 forward-backward message computation blocks, i.e., $\mathtt {FWBW\{p\}_{VNUCluster0}}$ with inputs, $\mathtt {inp\{p\}_{VNUCluster0}}$ and outputs, $\mathtt {op\{p\}_{VNUCluster0}}$ for $p = 0, 1, 2 \ldots , 9$ comprise the first stage of the pipeline. The second stage consists of the $\mathtt {VOut_0}$ computation block with input, $\mathtt {In_0}$ and output, $\mathtt {Out_0}$.

**Figure. 17:**
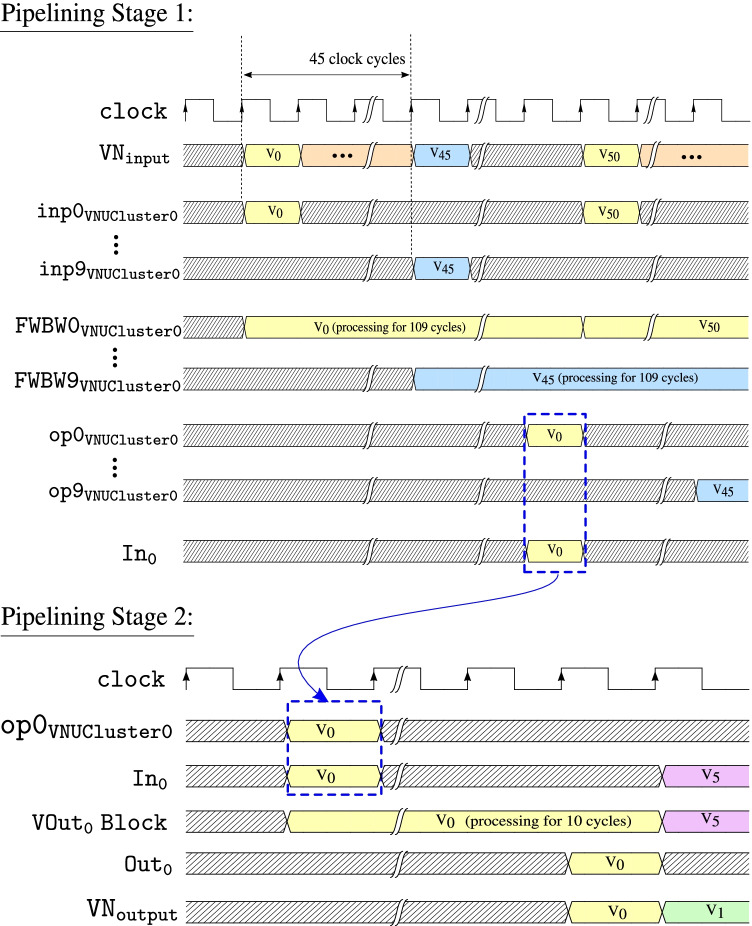
Timing diagram of $\mathtt {VNUCluster}$ block used in variable node message processing block of architecture C. The waveforms are shown particularly for $\mathtt {VNUCluster_0}$ block.

**Figure. 18:**
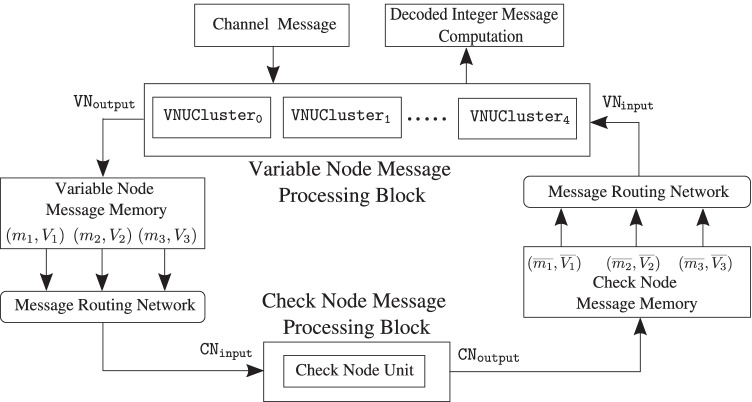
Top-level architecture for the LDLC decoder with a single check node and with two-stage pipelining to achieve an effective parallelism equivalent to 50 variable nodes.

Figure 17 shows the timing diagram for the various signals used in the two pipelining stages of the $\mathtt {VNUCluster_0}$ block. The resource requirements and throughput of the *variable node message processing block* used in architectures A, B, and C, are provided in Tables 1 and 2 respectively. Based on Tables 1 and 2, it is evident that parallelism and pipelining boost throughput of the *variable node message processing block* significantly. However, it is achieved at extra hardware cost. Figure 18 shows a high-level block diagram for decoder architecture C, that consists of a single check node and 5 $\mathtt {VNUCluster}$ blocks.

**Table 1:** Resource requirements of the variable node message processing block in architecture A, B and C.

Resource	Arch. A	Arch. B	Arch. C
ALM	8151	321128	406281
Dedicated Regs.	6464	146260	229380
DSPs	160	1509	1507

**Table 2:** Throughput (clock cycles/message) of the variable node message processing block in the architectures A, B and C.

Architecture	Throughput cycles/message
A	140
B	9.2
C	3.9

### Performance and Resource Usage

Decoder architectures A, B and C are implemented on an Intel FPGA (Arria 10, 10AX115N3F45I2SG) and the resource usage is provided in Table  3. All three architectures achieve the frame error rate shown in Fig. 6 at a clock frequency of 125 MHz. If the decoder is operated at a higher frequency, some critical paths in the design may have timing issues. Therefore, 125 MHz is the recommended fastest clock for our architectures in the target technology.

**Table 3:** Resource usage of different architectures

Resource	A 1 check node, 1 var. node	B 1 check node, 20 var. nodes	C 1 check node, parallelism equivalent to 50 var. nodes
ALMs (lut and reg)	12,560	328,490	411,436
Dedicated Registers	11,038	169,843	300,280
DSPs (27x27 mult.)	171	1,518	1,518
BRAMs	30	12	47

**Figure. 19:**
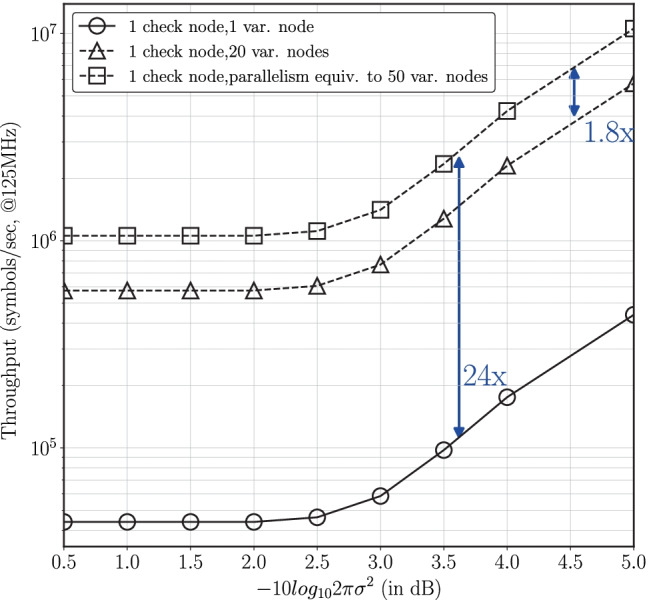
Throughput comparison of different decoder architectures for $n=1000$ and clock frequency of 125 MHz (modified from [18]).

Figure 19 shows the throughput comparison for these architectures. Architecture C attains a throughput of 10.5 Msymbols/sec at a distance of 5 dB from capacity which is a 24$\times$ improvement over the baseline implementation A and a 1.8$\times$ improvement over architecture B. Note that the decoder throughput varies over signal-to-noise-ratio (SNR) values due to early termination in the iterative decoding process.

To the best of our knowledge, there is no prior work on hardware implementation of LDLC decoders, and thus no direct comparator other than our previous paper [18]. Compared to [18], the work presented here achieves an overall improvement of 1.8$\times$ in decoding throughput over [18].

The storage requirement for this implementation is $O{(n\cdot d)}$ and the computational complexity is $O{(n\cdot d \cdot R)}$ where *n* is block length, *d* is degree for the LDLC design and *R* is number of the periodic extensions.

## Conclusion

This paper has described the performance results and design strategies used for a fixed-point single-Gaussian LDLC decoder implementation in hardware. After developing approaches to address the complexities of the hardware implementation, e.g., efficient approximations of the non-linear functions and a comprehensive quantization study, we have achieved a successful FPGA implementation of a decoder for low-density-lattice codes.

With the detailed knowledge gained from the serial and partially parallel single-Gaussian LDLC decoder implementations, this work can be extended to an LDLC decoder where messages exchanged are Gaussian mixtures. As an initial FPGA implementation of LDLC decoders, this work is key to future hardware implementations (FPGA or ASIC) of the LDLC decoders.
